# The Clinical Impact of Vascular Endothelial Growth Factor/Receptor (VEGF/R) Inhibitors on Voice

**DOI:** 10.1155/2023/1902876

**Published:** 2023-04-01

**Authors:** Christina Hui Lee Ng, Edward J. Damrose

**Affiliations:** ^1^Department of Otolaryngology Head and Neck Surgery, Sengkang General Hospital, Singapore; ^2^Division of Laryngology, Department of Otolaryngology Head and Neck Surgery, Stanford University Medical Center, Stanford, CA, USA

## Abstract

**Background:**

Vascular endothelial growth factor/receptor (VEGF/R) inhibitors are used in chemotherapy protocols to limit tumor angiogenesis. Recent evidence shows they are associated with hoarseness, but their impact on vocal cord function has not been fully identified.

**Objectives:**

To describe the preliminary laryngeal findings in patients undergoing chemotherapy with VEGF/R inhibitors, and to describe possible mechanisms of their effect on vocal fold function.

**Methods:**

A retrospective case series was conducted in a tertiary medical center between July 2008 and August 2022. Cancer patients developing hoarseness while undergoing chemotherapy with VEGF/R inhibitors underwent videolaryngostroboscopy.

**Results:**

The study included four patients. There were three females and one male, treated for breast, lung, and unknown primary cancer, respectively. All 4 patients developed hoarseness 2–7 days after initiating treatment with the VEGF/R inhibitor drugs aflibercept (*n* = 1) and bevacizumab (*n* = 3). In all patients, videolaryngostroboscopy revealed vocal fold bowing and pronounced glottic insufficiency. There were no signs of mucositis or paralysis. In three patients, treatment involved speech therapy, with or without vocal fold augmentation. The average follow-up was 10 months (range 8–12 months). In 2 patients, there was a return of normal voice quality with resolution of vocal fold bowing. In one patient, who remained on chemotherapy, there was persistent bowing.

**Conclusions:**

VEGF/R inhibitors are associated with vocal fold bowing and glottic insufficiency. This appears to be a reversible side effect. To our knowledge, this is only the second clinical description of the effect of VEGF/R inhibitors on vocal fold function.

## 1. Introduction

VEGF is a key factor in angiogenesis [1–7]. Through activation of the VEGF receptor (VEGF/R), it stimulates vascular endothelial cell proliferation and promotes endothelial cell survival [8, 9]. Aberrant angiogenesis has been implicated in cancer proliferation and metastasis [10]. Inhibition of VEGF/R signaling has emerged as a significant anticancer strategy.

With the increased use of VEGF/R inhibitors in chemotherapy protocols, consistent side effects have become apparent. Side effects such as hypertension [11, 12], hemorrhage [13], proteinuria, thrombosis, and poor wound-healing [14] are well documented. Recently, several case series have identified dysphonia as an additional complication [15, 16], which appears dose-related [17]. Only one study [18] has described the clinical appearance of the vocal folds following VEGF/R inhibitor therapy. The purpose of our study was to add to this growing body of literature by describing the laryngeal findings in patients undergoing chemotherapy with VEGF/R inhibitors and to discuss possible mechanisms for their effect on vocal fold function.

## 2. Methods

A retrospective case series was performed in a tertiary medical center between July 2008 and August 2022. Cancer patients who developed voice change during or closely following chemotherapy (less than 1 month) with VEGF/R inhibitors were included. Recordings of videolaryngostroboscopic examinations of each patient were reviewed. Vocal folds were evaluated for mucositis, hemorrhage, and mass lesions. During phonation, the vocal folds were evaluated for symmetry, amplitude, periodicity, mucosal wave, and glottic closure. Maximum phonatory time using sustained phonation of the vowel/e/was routinely recorded in our assessment, and the data were used for this case series.

Voice samples were obtained in a sound-treated room at comfortable loudness and pitch. Voice analysis was performed with the Kay/Pentax CSL (Computer Speech Laboratory) Model 4500 and the MDVP (Multi-Dimensional Voice Program). Patients were asked to sustain the vowel/a/for four seconds, and the following acoustic variables were measured: average fundamental frequency (F0), relative average perturbation (RAP), jitter, shimmer, noise-to-harmonic ratio (NHR), and voice turbulence index (VTI).

### 2.1. Statistical Analysis

Data analysis was performed using SPSS-22 for Windows (Statistical Package for the Social Sciences, SPSS Inc., Chicago IL, USA®). The variables were investigated using visual (histograms and probability plot) and analytical methods (Kolmogorov–Smirnov/Shapiro–Wilk test) to determine whether or not they were normally distributed. Categorical variables were interpreted by frequency tables. We performed analyses to describe and summarize the distributions of variables. The continuous variables were expressed as mean and standard deviation or as median and interquartile range, depending on the normality of their distribution. In two different periods of the disease, paired Student's *t*-test was used for variables with normal distribution. The statistically significant two-tailed*p* value was considered as *p* < 0.05.

## 3. Results

**Figure 1:**
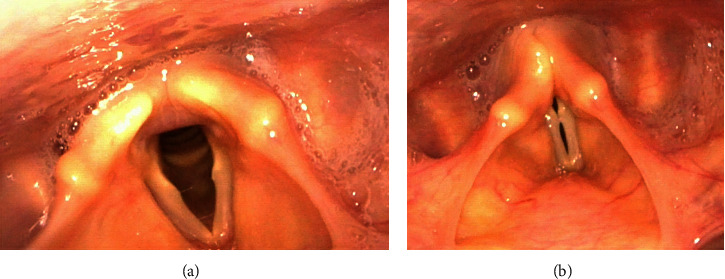
(a) Patient 4: vocal fold abduction. (b) Patient 4: vocal fold adduction. Midline glottic gap is noted.

Four patients (three females and 1 male, aged 45, 52, 65, and 64, respectively) were included in the study (Table 1). The primary diagnoses were lung, breast, lung, and colon cancer, respectively. None of the patients smoked or had preexisting laryngeal disease. They denied symptoms related to gastro-esophageal reflux, or reflux laryngitis. Patients 1, 2, and 4 received VEGF/R inhibitor bevacizumab, while patient 3 received aflibercept. All 4 patients developed dysphonia 2–7 days following the initiation of VEGF/R inhibitors. Video laryngostroboscopy revealed vocal fold bowing, a midline glottic gap, and glottic insufficiency in all patients. Photos of Patient 4 in full abduction and adduction are shown in Figures 1(a) and 1(b). The average maximum phonatory time (MPT) was 4.7 seconds (range 4 to 6 seconds). 3 patients underwent speech therapy. The average duration of dysphonia following initiation of VEGF/R inhibitor therapy was 6.7 months (range 4 to 10 months). The average follow-up was 10 months (range 8–12 months). In patients 2 and 3, there was a return of normal voice quality with resolution of vocal fold bowing. In patient 1, who remained on chemotherapy, there was persistent left-sided bowing. Patient 4 remains on VGEF/R inhibitor therapy and at this time is not interested in pursuing interventions. Acoustic analysis was included in the study (Table 2). The average jitter was 4.11% (range 3.03% to 5.19%). The average shimmer was 7.59% (range 5.98% to 9.23%). The average HNR was 26.75 (range 24 to 31). The average GRBAS score was 4.75 (range 4–6). The average VHI-10 score was 18.5 (range 13–22). In patients 2 and 3, there was return of normal parameters with the average maximum phonatory time (MPT) of 19s, average jitter 0.91%, average shimmer 2.46%, average HNR 18, GRBAS score 0, and average VHI-10 score of 4 after the end of VEGF/R inhibitor therapy.

## 4. Discussion

Abnormal angiogenesis, a hallmark of cancer proliferation, growth, and metastasis, is mediated by various proangiogenic factors. The VEGF pathway is one of the most important and best-studied angiogenic pathways. Inhibition of this pathway has emerged as a breakthrough in treating cancer patients [10]. Two common VEGF/R inhibitors used in conjunction with chemotherapy regimens are aflibercept and bevacizumab. Aflibercept is a protein comprised of segments of the human VEGF/R [19]. It functions as a decoy receptor for VEGF, thereby preventing VEGF from binding to its cell receptor and inhibiting tumor metastasis. Antitumor activity has been observed in ovarian carcinoma [20]. Bevacizumab is a monoclonal anti-VEGF antibody [21]. It prevents VEGF/R binding and inhibits the growth of tumor blood vessels. In conjunction with certain chemotherapy regimens, bevacizumab has demonstrated improved survival in colorectal cancer [11] and non-small-cell lung cancer [22, 23].

Voice change, hoarseness, and vocal fold palsy are well-documented side effects of several chemotherapy agents including vinca alkaloids [24] and cisplatin [25]. Recently, voice change has been observed in patients receiving chemotherapy treatment protocols that include VEGF/R inhibitor drugs [17, 18, 26]. In our small series, the dysphonia was secondary to bilateral vocal fold bowing with glottic insufficiency. In 2 patients, there was also evidence of mild vocal fold atrophy. The underlying pathophysiology for these clinical findings is not clear, but there are several possible mechanisms. First, VEGF is implicated in neuroprotection [27, 28]. Quattrini et al. showed the correlation between the severity of diabetic neuropathy and a decrease in VEGF [27]. They proposed that reduced VEGF levels might promote motor neuron degeneration by limiting neural tissue perfusion. It is possible that partial or selective recurrent laryngeal nerve denervation could produce transient vocal fold bowing with preserved mobility. Selective laryngeal EMG studies would be required to clarify this issue. Second, VEGF/R inhibitors might have an impact on vocal fold anatomy. Growth factors have been implicated as having a role in vocal fold lamina propria regeneration [29]. Hirano et al. demonstrated that injection of basic fibroblast growth factor (bFGF) to an aged and atrophic vocal fold resulted in improved mucosal wave and resolution of glottic insufficiency [29]. VEGF is known to stimulate collagen and elastin synthesis by smooth muscle [30]. It is possible that local changes in VEGF levels could influence the lamina propria characteristics and lead to vocal fold bowing and mucosal atrophy. Third, VEGF has been implicated in muscle regeneration. VEGF/R inhibitors have been reported to induce muscle weakness and pain [16]. Fourth, VEGF/R inhibitors may have a synergistic effect with other neurotoxic chemotherapy agents, such as cisplatin and paclitaxel, on neural dysfunction by inhibiting neural microvasculature function. Kirchmair et al. showed that VEGF gene therapy reversed cisplatin-induced neuropathy by restoring neural blood flow and peripheral nerve function [31]. Of note is that one of our patients (patient 1) presented with vocal fold hemorrhages. Bleeding and thromboembolic events have been associated with VEGF/R inhibitor treatment [16]. Fifth, VEGF/R inhibitors induce endothelial apoptosis with capillary regression in selected organs. Capillaries on larynx may be sensitive to VEGF/R inhibitors and undergo regression [32]. These proposed mechanisms are hypothetical with no studies proving the direct association between these clinical findings and patients receiving VEGF/R inhibitor drugs. A real association would only be possible with a prospective series or even in animal experiments.

In our small series, the vocal fold dysfunction induced by VEGF/R inhibitors appeared to be reversible. Although one patient (patient 2) required a vocal fold medialization, her bowing at the time of injection was unilateral and mild and significantly improved compared to her initial examination. Patient 1 remained on chemotherapy with persistent bowing, but in between each cycle of therapy, she reported voice improvement. We recognize that this is a small series, and further study is required to determine the reversibility of VEGF/R inhibitor therapy on voice quality.

The VEGF/R inhibitor drugs in this series were given in combination with other chemotherapy agents, which may cause voice change themselves [25]. However, we have not previously encountered the specific finding of vocal fold bowing in patients receiving chemotherapy protocols that did not include VEGF/R inhibitors. A larger prospective series with patients receiving solely VEGF/R inhibitors are required to confirm our observations.

## 5. Conclusion

Voice change has been reported as an adverse effect of chemotherapy treatment protocols with VEGF/R inhibitor drugs. However, to our knowledge, this is only the second clinical description of their effect on vocal fold function. The underlying pathophysiology, which may represent neuropathy, myopathy, or a loss of the lamina propria, requires further investigation.

## Figures and Tables

**Table 1:** Laryngeal findings following VEGF/R inhibitor therapy.

	G	Age	Diagnosis	Primary chemotherapy	VEGFI	Duration of therapy	Time to onset of dysphonia	Laryngeal findings	MPT (s)	Treatment for dysphonia	Duration of dysphonia	Follow-up	Outcome
1	F	45	Lung adeno-carcinoma	Erlotinib	Bevacizumab	10 months	2 days	BVF bowing	5	Speech therapy	10 months	10 months	Persistent bowing and dysphonia
2	F	52	Breast adeno-carcinoma	Capecitabine, paclitaxol	Bevacizumab	3 months	3 days	BVF bowing	5	Speech therapy	6 months	8 months	Normal vocal folds
3	M	64	Colon adeno-carcinoma	Cisplatin, pemetrexed	Aflibercept	3 months	7 days	BVF bowing	4	Speech therapy	4 months	12 months	Normal vocal folds
4	F	65	Lung adeno-carcinoma	Carboplatin, pemetrexed disodium	Bevacizumab	8 months	3 days	BVF bowing	6	Observation	8 months	9 months	Persistent bowing and dysphonia

BVF: bilateral vocal fold, VEGFI: vascular endothelial growth factor inhibitor.

**Table 2:** Acoustic analysis.

	G	Age	MPT (s)	Jitter (%)	Shimmer (%)	HNR	F0	GRBAS	VHI-10
1	F	45	5	3.025	5.982	25	158	5	22
2	F	52	5	4.255	7.895	27	170	4	18
			18	0.950	2.525	19	206	0	5
3	M	64	4	3.980	7.255	31	145	6	21
			20	0.865	2.388	17	120	0	3
4	F	65	6	5.185	9.230	24	175	4	13

## Data Availability

The retrospective data used to support the findings of this study are included within the article.

## References

[1] Asahara T., Murohara T., Sullivan A. (1997). Isolation of putative progenitor endothelial cells for angiogenesis. *Science*.

[2] Bates D. O., Heald R. I., Curry F. E., Williams B. (2001). Vascular endothelial growth factor increases Rana vascular permeability and compliance by different signalling pathways. *The Journal of Physiology*.

[3] Gerber H. P., McMurtrey A., Kowalski J. (1998). Vascular endothelial growth factor regulates endothelial cell survival through the phosphatidylinositol 3’-kinase/Akt signal transduction pathway: requirement for Flk-1/KDR activation. *Journal of Biological Chemistry*.

[4] Jain R. K. (2003). Molecular regulation of vessel maturation. *Nature Medicine*.

[5] Keck P. J., Hauser S. D., Krivi G. (1989). Vascular permeability factor, an endothelial cell mitogen related to PDGF. *Science*.

[6] Lamoreaux W. J., Fitzgerald M. E., Reiner A., Hasty K. A., Charles S. T. (1998). Vascular endothelial growth factor increases release of gelatinase A and decreases release of tissue inhibitor of metalloproteinases by microvascular endothelial cells in vitro. *Microvascular Research*.

[7] Senger D. R., Galli S. J., Dvorak A. M., Perruzzi C. A., Harvey V. S., Dvorak H. F. (1983). Tumor cells secrete a vascular permeability factor that promotes accumulation of ascites fluid. *Science*.

[8] Gille H., Kowalski J., Li B. (2001). Analysis of biological effects and signaling properties of Flt-1 (VEGFR-1) and KDR (VEGFR-2): a reassessment using novel receptor-specific vascular endothelial growth factor mutants. *Journal of Biological Chemistry*.

[9] Meyer M., Clauss M., Lepple-Wienhues A. (1999). A novel vascular endothelial growth factor encoded by Orf virus, VEGF-E, mediates angiogenesis via signalling through VEGFR-2 (KDR) but not VEGFR-1 (Flt-1) receptor tyrosine kinases. *The EMBO Journal*.

[10] Hsu J. Y., Wakelee H. A. (2009). Monoclonal antibodies targeting vascular endothelial growth factor: current status and future challenges in cancer therapy. *BioDrugs*.

[11] Hurwitz H., Fehrenbacher L., Novotny W. (2004). Bevacizumab plus irinotecan, fluorouracil, and leucovorin for metastatic colorectal cancer. *New England Journal of Medicine*.

[12] Veronese M. L., Mosenkis A., Flaherty K. T. (2006). Mechanisms of hypertension associated with BAY 43-9006. *Journal of Clinical Oncology*.

[13] Kamba T., McDonald D. M. (2007). Mechanisms of adverse effects of anti-VEGF therapy for cancer. *British Journal of Cancer*.

[14] Chen H. X., Cleck J. N. (2009). Adverse effects of anticancer agents that target the VEGF pathway. *Nature Reviews Clinical Oncology*.

[15] Goss G., Shepherd F. A., Laurie S. (2009). A phase I and pharmacokinetic study of daily oral cediranib, an inhibitor of vascular endothelial growth factor tyrosine kinases, in combination with cisplatin and gemcitabine in patients with advanced non-small cell lung cancer: a study of the National Cancer Institute of Canada Clinical Trials Group. *European Journal of Cancer*.

[16] Rixe O., Bukowski R. M., Michaelson M. D. (2007). Axitinib treatment in patients with cytokine-refractory metastatic renal-cell cancer: a phase II study. *The Lancet Oncology*.

[17] Drevs J., Siegert P., Medinger M. (2007). Phase I clinical study of AZD2171, an oral vascular endothelial growth factor signaling inhibitor, in patients with advanced solid tumors. *Journal of Clinical Oncology*.

[18] Hartl D. M., Ferte C., Loriot Y. (2010). Dysphonia induced by vascular endothelium growth factor/vascular endothelium growth factor receptor inhibitors. *Investigational New Drugs*.

[19] Chu Q. S. C. (2009). Aflibercept (AVE0005): an alternative strategy for inhibiting tumour angiogenesis by vascular endothelial growth factors. *Expert Opinion on Biological Therapy*.

[20] Moroney J. W., Sood A. K., Coleman R. L. (2009). Aflibercept in epithelial ovarian carcinoma. *Future Oncology*.

[21] Rosen L. S. (2002). Inhibitors of the vascular endothelial growth factor receptor. *Hematology-Oncology Clinics of North America*.

[22] Johnson D. H., Fehrenbacher L., Novotny W. F. (2004). Randomized phase II trial comparing bevacizumab plus carboplatin and paclitaxel with carboplatin and paclitaxel alone in previously untreated locally advanced or metastatic non-small-cell lung cancer. *Journal of Clinical Oncology*.

[23] Sandler A. B., Gray R., Brahmer J. (2005). Randomized phase II/III trial of paclitaxel (P) plus carboplatin (C) with or without bevacizumab (NSC # 704865) in patients with advanced non-squamous non-small cell lung cancer (NSCLC): an Eastern Cooperative Oncology Group (ECOG) Trial - E4599. *Journal of Clinical Oncology*.

[24] Whittaker J. A., Griffith I. P. (1977). Recurrent laryngeal nerve paralysis in patients receiving vincristine and vinblastine. *British Medical Journal*.

[25] Pomes A., Frustaci S., Cattaino G. (2009). Local neurotoxicity of Cisplatin after intra-arterial chemotherapy. *Acta Neurologica Scandinavica*.

[26] Saavedra E., Hollebecque A., Soria J. C., Hartl D. M. (2014). Dysphonia induced by anti-angiogenic compounds. *Investigational New Drugs*.

[27] Quattrini C., Jeziorska M., Boulton A. J., Malik R. A. (2008). Reduced vascular endothelial growth factor expression and intra-epidermal nerve fiber loss in human diabetic neuropathy. *Diabetes Care*.

[28] Storkebaum E., Lambrechts D., Carmeliet P. (2004). VEGF:once regarded as a specific angiogenic factor, now implicated in neuroprotection. *BioEssays*.

[29] Hirano S., Kishimoto Y., Suehiro A., Kanemaru S. J. (2008). Regeneration of aged vocal fold: first human case treated with fibroblast growth factor. *The Laryngoscope*.

[30] Heise R. L., Ivanova J., Parekh A., Sacks M. S. (2009). Generating elastin-rich small intestinal submucosa-based smooth muscle constructs utilizing exogenous growth factors and cyclic mechanical stimulation. *Tissue Engineering Part A*.

[31] Kirchmair R., Walter D. H., Ii M. (2005). Antiangiogenesis mediates cisplatin-induced peripheral neuropathy. *Circulation*.

[32] Hartl D. M., Bahleda R., Hollebecque A., Bosq J., Massard C., Soria J. C. (2012). Bevacizumab-induced laryngeal necrosis. *Annals of Oncology*.

